# The development of nurse-led clinics in China: Current status and future perspectives

**DOI:** 10.1097/MD.0000000000040527

**Published:** 2024-11-15

**Authors:** Xiaoshuang Lian, Weiming Qian, Yukun Zhang

**Affiliations:** a Department of Nursing, The Second Affiliated Hospital of Zhejiang University School of Medicine, Hangzhou, Zhejiang, China.

**Keywords:** advanced practice, current situation, development trend, nurse-led clinics, review

## Abstract

With the evolution of medical models and diverse healthcare service needs, nurse-led clinics (NLCs) have gained increasing attention. China began experimenting with NLCs in 1997 and their development has accelerated in recent years. This study reviews the current status, management practices, and innovative advancements in NLCs in China and compares them with those in other countries or regions. It analyses the existing challenges and obstacles to the development of NLCs in China, offering valuable insights for promoting their growth. Additionally, this study provides references that can guide the development of NLCs worldwide.

## 1. Introduction

Nurse-led clinics (NLCs) are an advanced nursing practice model. They are nurse-led healthcare service models that work independently or in collaboration with a multidisciplinary team to provide patients with health assessment, health education, consultation, treatment, psychological support, and case management.^[1]^ Studies have shown that opening NLCs plays a positive role in reducing patient mortality and promoting patient recovery.^[2–4]^ It promotes the development of the nursing discipline and further demonstrates the professional value of nurses. With the transformation of medical models and the diversified development of medical service needs, the development of NLCs is an inevitable trend to meet the various healthcare needs of patients.^[5,6]^

This study reviews the development, qualification access, and practice status of NLCs and analyzes the existing problems and obstacles of NLCs in China. It also describes the current status of the development of NLCs in other countries or regions, with the hope that this study can provide a reference for health administration departments and medical institutions as a basis for developing interventions to better implement NLCs.

## 2. Development status of NLCs

NLCs were first introduced in the United States in the 1960s. Initially, they were used in the field of chronic disease management to provide patients with health guidance and home-based rehabilitation programs. In the 1990s, NLCs entered the field of practice. NLCs have developed rapidly in the United States due to support from United States federal law in 2010, which clearly defined NLCs as primarily providing primary care services to underserved or disadvantaged groups.^[7]^ NLCs began to appear in the United Kingdom in the 1980s with the main purpose of reducing the burden on doctors, better responding to patients’ health service needs, and reducing medical costs. Currently, the United Kingdom has a relatively complete primary healthcare management policy.^[8]^ With the increasing improvement of Japan’s nursing higher education system and the successful implementation of the specialist nurse training program, NLCs began to emerge in Japan in the late 20th century with the aim of shortening the length of patients’ hospital stays and making healthcare family friendly.^[9]^ While the implementation of NLCs in Indonesia is poor, nurses only minimally implement outpatient care processes, resulting in poor patient satisfaction and safety.^[10]^

NLCs in China began late. Nursing consultation clinics focusing on maternal and child health care were first reported in 1997.^[11]^ Reports of colostomy NLCs and nursing practices appeared in 2003,^[12]^ and the literature related to NLCs increased significantly, showing a steady upward trend after 2013. Previous studies have mainly focused on chronic diseases.^[13]^ Although NLCs are gradually being conducted in various provinces in China, research related to NLCs in China is still in the preliminary exploration stage.

## 3. Current status of management of NLCs

### 3.1. Personnel qualification

A study conducted in 2017 on the current practice status of NLCs nationwide showed that 80% of 1181 NLC nurses had the title of chief nurse or above, 90% had over 10 years of work experience, and 80% had a bachelor’s degree. More than 3% of the participants had a postgraduate degree.^[14]^ A survey on the establishment and current situation of NLCs in tertiary hospitals found that among the 2701 nurses attending nursing specialty outpatient clinics, 81.2% had a bachelor’s degree or above, 75.9% had the title of chief nurse or above, and 55.28% had obtained a professional health qualification from the administrative department. In addition, 55.2% had obtained specialist certification from relevant academic groups.^[15]^ To standardize the management of NLCs, the “Construction of Management Standards for Nurse-led Clinics in Guangdong Province” suggests that nurses in clinics should meet the following requirements in terms of education and work experience: a full-time undergraduate degree or above, the title of chief nurse or above, and work experience of ≥10 years; or a master’s degree or above, with ≥3 years of work experience.^[16]^ At present, different hospitals have varying requirements for nurses in NLCs, and some hospitals have relatively low thresholds. For example, a nationwide survey on the opening of peripherally inserted central catheter NLCs found that 45.1% of hospitals allowed work in NLCs by passing hospital internal assessments and 2.1% permitted such work without requiring certification.^[17]^ Overall, NLCs need to have a bachelor’s degree or above, senior qualifications, rich clinical experience in specialized nursing, the title of chief nurse or above, and a specialized nursing certificate.

In the 1990s, nursing clinics began to emerge in Hong Kong, providing continuity of professional treatment services for critically ill and chronically ill patients.^[18]^ NLCs must be reviewed and certified by the Hong Kong Hospital Authority. Nurse practitioner (NP) are the only advanced nursing professionals in Taiwan’s nursing industry who have a national license. Most NPs serve acute medical institutions to solve the problem of insufficient doctors.^[19]^ NPs mainly provide continuous and integrated nursing and medical care, together with doctors.

Specialized Japanese nursing talents are divided into certified nurse specialists (CNS) and certified nurses (CN). Nursing clinics require nurses to have rich practical experience in the corresponding specialized field. Owing to differences in personnel allocation among hospitals, there is currently no unified regulation on the job requirements of nursing clinics. Overall, the CNS and CN are the main requirements. Therefore, nursing clinics are one of the most important places where the CNS and CN are active.^[20]^ In Singapore, nursing specialty clinics are typically conducted by advanced practice nurses in collaboration with doctors and other healthcare professionals to assess, diagnose, and manage patients in areas such as primary care, geriatric care, and mental health.^[21]^ Advanced practice nurses are legally limited to registered nurses who have completed recognized Master of Nursing programs, with collaborative prescription rights and limited referral and admission privileges.

### 3.2. Types of NLCs

According to the literature review, at present, NLCs in China mainly include PICC nursing clinics,^[17]^ wound stoma nursing clinics,^[22]^ diabetes nursing clinics,^[23]^ chronic heart failure nursing clinics,^[24]^ implantable pacemaker follow-up,^[25]^ chronic obstructive pulmonary disease nursing clinics,^[26]^ Parkinson disease nursing clinics,^[27]^ urology nursing clinics,^[28]^ lymphedema nursing clinics,^[29]^ stroke nursing clinics,^[30]^ swallowing disorder nursing clinics,^[31]^ clinical nutrition support nursing clinics,^[32]^ respiratory nursing clinics,^[33]^ inflammatory bowel disease nursing clinics, cognitive training, psychiatry, and health management.^[34]^ Traditional Chinese medicine (TCM) nursing clinics,^[25]^ childhood asthma, epilepsy, constipation, and intestinal canal nursing clinic.^[35,36]^ Midwife nursing clinics, maternal and child nursing clinics,^[37]^ and comprehensive nursing clinics, etc.^[38]^

From the perspective of hospital categories, NLCs covered general hospitals, TCM hospitals, psychiatric hospitals, and maternal and child specialist hospitals. From the perspective of hospital levels, those reported in the literature are all second-level or above hospitals. From the form of development, there are currently 2 main types: independent nursing specialty clinics and medical-nursing collaborative nursing specialty clinics in China. From a functional perspective, they can be divided into treatment, health consultation, and comprehensive nursing clinics.

In America, NLCs encompass primary care, chronic disease management (diabetes, hypertension), mental health, women’s health, pediatrics, geriatrics, and urgent care. Nurses in these clinics have the right to practice independently or collaboratively, prescribe medications, and manage patient care autonomously, depending on state regulations.^[39]^ In the United Kingdom, NLCs cover various specialties, including chronic disease management (diabetes, asthma, hypertension), minor illness and injury treatment, sexual health, wound care, and smoking cessation. These clinics aim to provide accessible and specialized care, reducing the burden on general practitioners and hospitals.^[40]^

NLCs in Hong Kong follow the British healthcare system and have gradually extended from the community to various specialist fields, such as orthopedics, cardiovascular care, cancer care, rheumatic diseases, and geriatric care (cognitive impairment), stroke, wound ostomy, psychiatry, diabetes specialist care, etc.^[41,42]^ However, only NPs have partial prescription rights. The classification of specialized nursing in Hong Kong is more detailed. In addition to the pain specialty with most practitioners, there are also specialties in stroke, hyperlipidemia, hyperglycemia, and hypertension, as well as the physical heritage specialty for handling patients with urinary and fecal incontinence.^[21]^

NLCs in Japan cover a wide range of areas such as diabetes care, stoma care, midwifery, cancer care consultation, chemotherapy, lymphedema, urinary incontinence, peritoneal dialysis, home oxygen therapy, pediatric care, cognitive disorders, infection counseling, smoking cessation, and lifestyle diseases. Diabetes care (foot care) clinics had the highest rate of availability (58.4%), followed by stoma care clinics (54.5%), midwifery clinics (30.7%), and cancer care counseling clinics (27.5%).^[43]^ Since the development of nurse specialists in Singapore in 1993, there have been a number of NLC areas, mainly including psychiatric nursing, cardiovascular disease nursing, rheumatology nursing, diabetes mellitus, geriatric care (cognitive impairment), ostomy care, community care, etc.^[44–46]^

In summary, in terms of style, there are health care cooperative clinics and nurse-independent clinics both at home and abroad. In terms of mode, China is mainly in the nursing accountability mode, while other countries are mainly in staged functional care. In terms of authority, specialist nurses have certain prescribing rights in countries such as America, England, Singapore, Hong Kong, etc, whereas in China, only some areas have very limited prescription rights.

### 3.3. NLCs service content

The services provided by nursing specialty clinics in China mainly include PICC care, including PICC insertion and maintenance, complication management, and health education; various acute and chronic wound treatments, including burns and scalds, postoperative dressing changes and suture removal, and surgery; treatment and health education of wounds, such as posterior incision infections, pressure injuries, diabetic foot, lower limb arteriovenous ulcers, cancerous wounds, and drug-resistant bacterial infections; related disease assessment and evaluation; health consultation; direct care; individualized health education guidance; psychological support; self-management skills guidance (rehabilitation guidance); operating standard examples; complication screening; patient home care, including wounds of home patients, ostomy care, PICC care, drainage tube care, etc; and in-hospital and out-of-hospital nursing consultation.

NLCs in China are guided by the holistic nursing philosophy. In terms of categories, these include services such as education, counseling, therapeutic treatment, and case management. In terms of dimensions, they encompass 3 aspects: somatic, psychological, and social. In terms of the form of follow-up, in mainland China, it is mainly conducted through telephone, outpatient clinics, and WeChat groups.

In Hong Kong, 80% of nursing clinics are for rehabilitation care, such as professional assessment of patients with diabetes mellitus, heart disease, and stroke, and 20% are for wound care and care of patients with chronic pain.^[47]^ In Taiwan, nurses perform both invasive and noninvasive medical operations under the supervision of physicians.^[19]^ (1) The invasive scope includes wound, pipeline, examination, and other treatments. (2) The noninvasive scope includes prescribing forms required for specific medical procedures, preliminary judgment of tests and examinations, noninvasive medical treatments, and related medical consultations.

### 3.4. Government policy support

To further standardize the management of NLCs, the Shanghai Municipal People’s Government issued the “Notice on Further Standardizing the Management of Nursing Specialty Outpatient Clinics” in January 2023^[48]^ which clearly stipulated the content of nurse-led service projects and requirements for NLCs nurses. In September 2023, the Shenzhen Municipal Health Commission and Shenzhen Medical Insurance Bureau jointly issued “Shenzhen Specialized Nurse Training and Management Measures,”^[49]^ which unified the admission standards for specialized nurses and clarified their job responsibilities in professional activities. Specialized nurses can perform relevant professional activities in NLCs or community health service institutions, and their prescriptions can be included in medical insurance payments. This is the first local government to issue regulations in this regard and the first in the country to legislate to give specialist nurses the right to prescribe, although only some external medicines can be prescribed. The promulgation of the “Measures” provided a reference for other places across the country to carry out specialist nurse training management.

### 3.5. NCLs charges

Most hospital specialist care clinics currently use registration fees for general outpatient clinics, with costs varying by province. There are even some free nursing clinics. Shanghai clearly mentioned in the “Notice on Further Standardizing the Management of Nursing Specialty Outpatient Clinics” that NLCs in medical insurance-designated medical institutions are charged according to the general outpatient examination fees and are included in the scope of medical insurance payment. In addition, it is necessary to optimize the medical treatment process continuously. For projects that are assessed to need to provide continuous wound care services, a single registration fee should be covered for one course of treatment (3–5 times), and the patient’s medical burden should not be increased. The “Shenzhen Specialist Nurse Training and Management Measures” stipulates in the professional standards chapter that the charging standards for examination application forms, treatment application forms, and external drugs issued by specialist nurses should be in accordance with the relevant regulations on medical service charges.

## 4. Innovative nursing service models of NCLs

In recent years, some hospitals have begun to explore the construction of shared nursing outpatient services (shared outpatient services refer to group outpatient services led by specialist nurses, which carry out continuous medical visits to patients in a supportive group environment). For example, the Affiliated Hospital of Integrated Traditional and Western Medicine of Nanjing University of Chinese Medicine has established a shared outpatient service led by specialist nurses for patients with type 2 diabetes, effectively reducing waist circumference, total cholesterol, and triglycerides in type 2 diabetes patients, by developing shared outpatient management content, sharing outpatient personal visits, and developing individualized goals including blood sugar, blood pressure, blood sugar monitoring, diet, exercise, medication, etc, improving self-management behavior and self-management efficiency of patients, and improving the quality of life of patients.^[50]^ Wuhan Central Hospital Affiliated to Tongji Medical College of Huazhong University of Science and Technology is guided by patient needs and has established a comprehensive nursing clinic that integrates multiple specialist nursing services.^[38]^ The hospital packages patient needs and breaks the traditional single nursing specialist outpatient service model to provide patients with nursing services, including wound stoma care, PICC care, diabetic foot care, drainage tube care, etc, reducing the need for patients with diverse needs to shuttle between various clinics, simplifying the medical treatment process, shortening waiting time, and improving the patient’s medical experience. The First Affiliated Hospital of Fujian Medical University has opened a nursing and treatment center since 2021, and has set up NLCs such as intravenous treatment, wound treatment, stoma care, comprehensive respiratory treatment, maternal and infant care, etc; patients only need to register for a single clinic and can get a “one-stop” solution for a number of care issues.

From a model perspective, after the country issued the “Internet + Nursing Service” Pilot Work Plan in 2019, some provinces actively explored new “Internet + Nursing” service models and adopted an “online application, offline service” model to provide continuing care services to discharged patients or special groups suffering from diseases and limited mobility.^[51,52]^ In addition, “online consultation” based on “Internet + Nursing” has increasingly become an emerging model, that is, patients can find nursing experts for corresponding diseases through mobile APPs to meet patients’ needs for perioperative care and daily health guidance for diseases. It can meet multifaceted medical needs, reduce the number of patients’ trips to the hospital, and improve the patient’s medical experience.

Additionally, TCM nursing clinics have Chinese characteristics. TCM NLCs are an advanced nursing practice model with Chinese characteristics that are based on the overall concept of TCM and syndrome differentiation and care and apply TCM nursing technology to provide disease prevention and health care. The visiting nurse establishes the patient’s health file based on the 4 diagnostic methods of TCM (observation, smell, questioning, and palpation), implements nursing operation techniques with TCM characteristics (such as Guasha, auricular pressure, cupping, and moxibustion) based on the nursing problems derived from syndrome differentiation analysis, and applies the theoretical knowledge of TCM to provide health care, dietary nutrition, emotional adjustment, and meridian health guidance.^[53]^

## 5. Main problems

### 5.1. Exploration of operational mechanisms

A clear regulatory framework is needed to define the scope of practice and establish licensing and accreditation standards. There is a lack of national regulations on the management of NLCs and nationally unified qualification certification for nurses practicing in nursing outpatient clinics, there are even no clear provisions on the work scope of outpatient nurses, job roles and places of practice, and so on. In addition, pricing and charging standards for nursing outpatient clinics are not yet perfect. Most nursing clinics do not charge registration fees and can only charge according to service items, making it difficult to reflect the professional value of nursing work and reducing the motivation of outpatient nurses to work.

### 5.2. Obstacles to the implementation of nurse prescription rights

The scope of authority of NLCs is limited, and there are fewer implementations of nurse prescription rights. As of 2023, only Shenzhen City in China has implemented nurse prescription rights. At present, there are three major obstacles to nurses’ prescription rights in China: the environment, policies, and personnel. The location of the consultation is limited, the form is relatively single, and there is a significant gap in the development levels between regions.

### 5.3. Limitations and challenges in the establishment and utilization of NCLs

The establishment of NCLs in China is mostly limited to hospitals and does not cover communities; most of them are patients who come to the hospital for treatment, lacking forms such as family follow-up. In addition, the types of nursing specialty clinics are mostly for chronic disease management, which is not conducive to popularizing basic health care and building a continuous nursing service model. According to a survey^[15]^ of 330 tertiary hospitals in 19 provinces of China in 2017, China opened 926 nursing clinics, but most of the hospitals are located in economically developed East China and pilot provinces and cities for medical reform. Highly educated nursing graduates have not yet been fully utilized.

With the establishment of a professional master’s degree in nursing and its becoming the important training type, China’s nursing talent reserve is growing day by day. However, in clinical practice, master’s degrees in nursing have not fully utilized advanced practical skills, and the training and certification of specialized nurses lack a unified and perfect mechanism. Nursing talents have not fully realized their own values, and there is a phenomenon of separation between training and application. At present, many hospitals prioritize training over utilization, which means investing considerable manpower, material resources, and financial resources to cultivate the various outpatient abilities of nursing staff. However, after training, most nursing staff still return to the inpatient department and have not developed in the outpatient direction, resulting in separation between training and utilization.

#### 5.3.1. Cultural barriers

The general public often views nurses as support staff rather than primary caregivers. This perception limits the acceptance and trust in NLCs, as people may prefer to see doctors for their healthcare needs. Besides, there is limited public awareness and understanding of the role of NPs. Patients may prefer seeing doctors due to a lack of trust in nurses’ capabilities to provide primary and specialized care. What’s more, within the nursing profession, there might be resistance to taking on expanded roles due to insufficient training or fear of professional.

### 5.4. Challenges in funding and awareness for specialized nursing clinics

Securing sufficient funding and resources, including insurance coverage for services, is vital for sustainability. The promotion efforts are limited, and the awareness level of medical staff and the general public towards specialized nursing clinics is relatively low, resulting in low patient visits and difficulty in achieving results in some hospitals. This has led to a certain degree of waste of nursing clinic resources.

## 6. Implications for nursing and health policy

### 6.1. Conduct a national survey

It has been over 7 years since the last national survey of NLCs was conducted. Therefore, it is recommended that policymakers conduct timely surveys or sampling studies on the current practice status of NLCs nationwide, understand the current status of personnel qualifications, service types, service content, etc in nursing specialty clinics in various provinces, and establish a unified national nursing outpatient management standard based on the national survey so as to be included in regulatory management.

### 6.2. Expand scope of practice

Enact national regulations to empower NPs. This policy will allow them to prescribe medications, make diagnoses, and manage patient care independently. Implement standardized national guidelines, along with strict entry standards and training mechanisms to ensure nurses’s competence. This initiative aims to improve the efficiency and effectiveness of the healthcare system by fully tapping the potential of NPs and increasing access to healthcare services, especially in rural and underserved areas.

### 6.3. Clarify the charging standards

The traditional performance evaluation system cannot adapt to the working mode of nursing clinics, and lower fees do not reflect the service value of specialist nurses. It is recommended that the country explore performance evaluation methods suitable for NLC nurses, reflect nursing values, and mobilize nurses’ work enthusiasm.

### 6.4. The government needs to strengthen guidance and publicity

Each provincial government department should work together with the health administrative management department to strengthen organizational leadership, create favorable conditions, increase publicity efforts for nursing clinics, launch national campaigns to educate the public about the role and benefits of NLCs, and continuously improve the utilization rate of nursing clinics.

## 7. Conclusion

China has a large population base and an imbalance in the supply and demand of medical resources, and the aging of the population continues to deepen and accelerate, increasing the incidence of chronic diseases.^[54]^ The establishment and development of NLCs can alleviate some of the pressure on medical supply and demand, save medical resources, can enhance access and quality of care for patients, offer professional growth and leadership opportunities for nurses, and improve system efficiency and cost-effectiveness for policymakers. Compared to some developed countries, the development of foreign NLCs is relatively mature and has formed unified and complete management standards. The development level of NLCs in China is uneven, practice locations and nurses’ prescribing rights are limited, and there is still a lack of unified management standards in the exploratory stage.

To meet the needs of diverse care services, it is urgent to establish a sound NLCs management system, effectively implement mid- and long-term training and development plans for nursing talent, promote TCM NLCs with Chinese characteristics, and at the same time explore new models of diversified nursing specialty outpatient services to promote further development of NLCs in China. It also provides ideas for the development of nursing clinics in other countries worldwide.

## Author contributions

**Writing – original draft:** Xiaoshuang Lian, Weiming Qian.

**Writing – review & editing:** Yukun Zhang.

## References

[1] Molassiotis A Liu XL Kwok SW. Impact of advanced nursing practice through nurse-led clinics in the care of cancer patients: a scoping review. Eur J Cancer Care (Engl). 2021;30:1. 10.1111/ecc.13358 33169476

[2] Randall S Crawford T Currie J River J Betihavas V. Impact of community based nurse-led clinics on patient outcomes, patient satisfaction, patient access and cost effectiveness: a systematic review. Int J Nurs Stud. 2017;73:24–33. 28531549 10.1016/j.ijnurstu.2017.05.008

[3] Corones-Watkins K Cooke M Theobald K. Effectiveness of nurse-led clinics in the early discharge period after percutaneous coronary intervention: a systematic review. Aust Crit Care. 2021;34:510–7. 33272768 10.1016/j.aucc.2020.10.012

[4] Pouresmail Z Heshmati Nabavi F Valizadeh Zare N. Outcomes of patient education in nurse-led clinics: a systematic review. J Caring Sci. 2023;12:188–200. 38020736 10.34172/jcs.2023.31891 PMC10663435

[5] Lin S Xiao L Chamberlain D. Nurse-led health coaching programme to improve hospital-to-home transitional care for stroke survivors: a randomised controlled trial. Patient Educ Couns. 2022;105:917–25. 34294494 10.1016/j.pec.2021.07.020

[6] Whitehead P Frechman E Johnstone-Petty M. A scoping review of nurse-led advance care planning. Nurs Outlook. 2022;70:96–118. 34627618 10.1016/j.outlook.2021.08.002

[7] Chan RJ Marx W Bradford N. Clinical and economic outcomes of nurse-led services in the ambulatory care setting: a systematic review. Int J Nurs Stud. 2018;81:61–80. 29518623 10.1016/j.ijnurstu.2018.02.002

[8] Loftus LA Weston V. The development of nurse-led clinics in cancer care. J Clin Nurs. 2001;10:215–20. 11820342 10.1046/j.1365-2702.2001.00488.x

[9] Tateishi M Nakanishi K Takehara K Honda I Yamauchi T. Nursing activities at clinics in rural areas in Japan: gaps between recognition of importance and implementation. Nagoya J Med Sci. 2020;82:251–60. 32581405 10.18999/nagjms.82.2.251 PMC7276418

[10] Zendrato MV Hariyati RTS Afifah E. Outpatient nursing care implementations in Indonesian regional public hospitals. Enferm Clin. 2019;29:449–54.

[11] Feng Y Cai Y Sun M. Experience in setting up nursing consultation clinics. Chin J Nurs. 1997;2:115–6.

[12] Ding Y. Practice of setting up specialized outpatient clinics for colostomy nursing. J Qilu Nurs. 2003;9:397–8.

[13] Niu D Li S Zhu X. Bibliometric and content analysis of the development status of nursing outpatients in China. Chin J Prac Nurs. 2017;33:2389–93.

[14] Ding S Huang J Li T. A survey on the current practice status of nurses in nursing specialty outpatient clinics. Chin Nurs Manag. 2017;17:1302–6.

[15] Gao FL Ding S Huang J. A survey and analysis of the current situation of the establishment and practice of nursing specialty clinics in tertiary hospitals in China. Chin Nurs Manag. 2017;17:1297–302.

[16] Fan F Lin W Yan F Zhou X. Construction of management standards for specialized nursing outpatients in Guangdong province. Chin J Nurs. 2020;55:1217–23.

[17] Nie S Wang L. A survey and study on the establishment of PICC specialized nursing clinics in 743 hospitals nationwide. Chin J Mod Nurs. 2019;25:3728–32.

[18] Pun J Tsang C Wong J Kong B. Experience of patients with rheumatoid arthritis: a qualitative study of a nurse-led clinic. Clin Nurs Res. 2023;32:840–9. 36999603 10.1177/10547738231164395

[19] Lin CH Siao SF Tung HH Chung KP Shun SC. The gaps of healthcare service quality in nurse practitioner practice and its associated factors from the patients’ perspective. J Nurs Scholarsh. 2021;53:378–86. 33634957 10.1111/jnu.12641

[20] Song P Tang W. The community-based integrated care system in Japan: health care and nursing care challenges posed by super-aged society. Biosci Trends. 2019;13:279–81. 31327797 10.5582/bst.2019.01173

[21] Xu C Xie H Zhou Z Govindasamy A Mao R Chan YH. Advanced practice nurses led clinic in a psychiatric hospital: an outcome evaluation in Singapore. Arch Psychiatr Nurs. 2020;34:129–33. 32513462 10.1016/j.apnu.2020.03.003

[22] Yang D Zhang R Kirkland-Kyhn H. Training and practice of wound ostomy continence nurse specialists in China. Wound Manag Prev. 2023;69:28–31. 38052013

[23] Wang Q Shen Y Chen Y Li X. Impacts of nurse-led clinic and nurse-led prescription on hemoglobin A1c control in type 2 diabetes: a meta-analysis. Medicine (Baltim). 2019;98:e15971. 10.1097/MD.0000000000015971 PMC6571354 31169727

[24] Qiu X Lan C Li J Xiao X Li J. The effect of nurse-led interventions on re-admission and mortality for congestive heart failure: a meta-analysis. Medicine (Baltim). 2021;100:e24599. 10.1097/MD.0000000000024599 PMC7899814 33607793

[25] Dong Z Wei L Sun X. Experiences of nurses working in nurse-led clinics in traditional Chinese medicine hospitals: a focused ethnographic study. Nurs Open. 2023;10:603–12. 36054474 10.1002/nop2.1326 PMC9834534

[26] Chen L. Application of chronic obstructive pulmonary disease nursing specialist clinic in pulmonary rehabilitation training for stable chronic obstructive pulmonary disease patients. Chin Mod Med. 2021;28:213–16.

[27] Lu L. Evaluation of the establishment and implementation of specialized nursing clinics for Parkinson’s disease. Chin Foreign Med Res. 2020;18:100–02.

[28] Ma Z Niu M Qian C Ding Y Gui W. Research progress on the establishment of specialized nursing outpatient positions and its inspiration for the construction of urology nursing outpatient clinics. Mod Clin Nurs. 2023;22:93–8.

[29] Wang X Wang J Xia Y He X Zhou X. Practice and reflection on establishing outpatient services in medical technology, pharmacy, and nursing. Chin Hosp. 2022;26:94–6.

[30] Fan P Li H Li L Ruan H Chen M. Establishment and practice of post-stroke cognitive impairment care clinic. Chin J Nurs. 2018;53:1352–5.

[31] Guo Y Guo L Dong X. Nursing adherence, barriers and facilitators to conduct post-stroke dysphagia screening and assessment: a study based on theoretical domain framework. J Clin Nurs. 2023;32:3787–96. 36717977 10.1111/jocn.16623

[32] Huang Y Wang X Li P Peng N. The establishment and implementation effect of clinical nutrition support nursing outpatient clinic. Parenter Enteral Nutr. 2016;23:382–4.

[33] Cai X Guo L. Practice and exploration of establishing specialized respiratory nursing clinics. Chin J Geriatr Care. 2013;11:111–2.

[34] Pan Y Chen R. The effect of a nurse-led cognitive behavioral protocol on depressive symptoms and coping strategies of dementia caregivers. J Nurs Res. 2019;27:e55. 31107775 10.1097/jnr.0000000000000327

[35] Yu X Li C Hu L. The construction and operation of specialized nursing clinics for children with epilepsy. J Nurs sci. 2022;37:50–3.

[36] Chen J Cai D Han C Xu T. The establishment and practice of specialized nursing clinics for children with constipation. J Nurs. 2022;29:17–20.

[37] Lin G. Analysis of intervention effects of maternal and infant specialized nursing clinics on pregnant women with gestational hypertension. Prev Treatment Cardiovasc Dis. 2022;12:38–40.

[38] Teng L Xu M Xiao Y. The establishment and practice of a comprehensive nursing clinic led by specialized nurses. J Nurs. 2023;30:37–40.

[39] McMenamin A Turi E Schlak A Poghosyan LA. A systematic review of outcomes related to nurse practitioner-delivered primary care for multiple chronic conditions. Med Care Res Rev. 2023;80:563–81. 37438917 10.1177/10775587231186720 PMC10784406

[40] Connolly C Cotter P. Effectiveness of nurse-led clinics on healthcare delivery: an umbrella review. J Clin Nurs. 2023;32:1760–7. 34970816 10.1111/jocn.16186

[41] Lim LL Lau E Ozaki R. Team-based multicomponent care improved and sustained glycaemic control in obese people with type 2 diabetes (T2D) in a diabetes centre setting: a quality improvement program with quasi-experimental design. Diabetes Res Clin Pract. 2022;194:110138. 36328212 10.1016/j.diabres.2022.110138

[42] Wong A Kwok V Wong F. Improving post-acute stroke follow-up care by adopting telecare consultations in a nurse-led clinic: study protocol of a hybrid implementation-effectiveness trial. J Adv Nurs. 2024;80:1222–31. 37950400 10.1111/jan.15960

[43] Noguchi M Kojima S Sairenchi T. Japan trial in high-risk individuals to enhance their referral to physicians (J-HARP)—a nurse-led, community-based prevention program of lifestyle-related disease. J Epidemiol. 2020;30:194–9. 30982808 10.2188/jea.JE20180194 PMC7064550

[44] Wang W Seah B Jiang Y. A randomized controlled trial on a nurse-led smartphone-based self-management programme for people with poorly controlled type 2 diabetes: a study protocol. J Adv Nurs. 2018;74:190–200. 28727183 10.1111/jan.13394

[45] Saxena N George PP Teo KW. Evaluation of an integrated primary care-led dementia shared care program in Singapore: an effectiveness and cost-effectiveness study. Geriatr Gerontol Int. 2018;18:479–86. 29193721 10.1111/ggi.13196

[46] Woo BFY Tam WWS Rangpa T Liau WF Nathania J Lim TW. A Nurse-led Integrated Chronic care E-enhanced Atrial Fibrillation (NICE-AF) clinic in the community: a preliminary evaluation. Int J Environ Res Public Health. 4467;19:4467. 35457336 10.3390/ijerph19084467 PMC9026946

[47] Cheng HY Chair SY Wang Q Sit JW Wong EM Tang SW. Effects of a nurse-led heart failure clinic on hospital readmission and mortality in Hong Kong. J Geriatr Cardiol. 2016;13:415–9. 27594868 10.11909/j.issn.1671-5411.2016.05.013 PMC4984577

[48] Shanghai Medical Security Bureau and Shanghai Health Commission. Notice on further standardizing the management of nursing specialty outpatient clinics. 2023;2024(2024-01-25).

[49] Shenzhen Medical Security Bureau Shenzhen Health Commission. Shenzhen specialist nurse training and management measures. 2023;2024(2024-01-25).

[50] Li DD Yao Y Cao L. Construction and application of shared outpatient service for type 2 diabetes patients led by specialized nurses. Chin J Nurs. 2022;57:17–22.

[51] Tian F Xi Z Ai L. Investigation on nurses’ willingness to “Internet + Nursing Service” and analysis of influencing factors. J Multidiscip Healthc. 2023;16:251–60. 36726482 10.2147/JMDH.S396826 PMC9885965

[52] Yan W Liu L Huang WZ. Study on the application of the Internet + Nursing service in family rehabilitation of common bone and joint diseases in the elderly. Eur Rev Med Pharmacol Sci. 2022;26:6444–50. 36196694 10.26355/eurrev_202209_29743

[53] Wei P Zhang Y Wu S. Current situation and influencing factors of traditional Chinese medicine nursing clinic in Henan Province. J Healthc Eng. 2022;2022:8941922. 35356611 10.1155/2022/8941922 PMC8959967

[54] Xia C Chen W. Fractions and trends of cancer burden attributable to population ageing in China. Zhonghua Zhong Liu Za Zhi. 2022;44:79–85. 35073652 10.3760/cma.j.cn112152-20211012-00756

